# Automated Detection of Parasitic Elements in Veterinary Fecal Samples Using a Deep Learning-Based Object Detection Framework

**DOI:** 10.3390/vetsci13030257

**Published:** 2026-03-10

**Authors:** Jing Yang, Bo Yang, Qingxiang You, Zhenqing Li, Yoshinori Yamaguchi

**Affiliations:** 1 Faculty of Engineering, Anhui Sanlian University, Hefei 230601, China; yangjing@mail.slu.edu.cn; 2 School of Optical-Electrical and Computer Engineering, University of Shanghai for Science and Technology, Shanghai 200093, China; 3 School of Computer Science and Information Engineering, Changzhou Institute of Technology, Changzhou 213032, China; 4 Comprehensive Research Organization, Waseda University, Tokyo 162-0041, Japan; yoshi.yamaguchi@ap.eng.osaka-u.ac.jp

**Keywords:** YOLOv8, *Spirometra* eggs, *Dipylidium* egg packets, hookworm eggs, *Ascaris* eggs, *Giardia* cysts, *Trichomonas* trophozoites

## Abstract

Parasitic infections in veterinary medicine are commonly diagnosed through microscopic examination of fecal samples, yet traditional manual methods are labor-intensive and subject to diagnostic variability. This study investigates YOLOv8 for automated identification of parasitic elements in fecal microscopy images. Six parasitic taxa were analyzed at 1000×, 2500×, and 10,000× magnifications: *Spirometra* eggs, *Dipylidium* egg packets, hookworm eggs, *Ascaris* eggs, *Giardia* cysts, and *Trichomonas* trophozoites. The dataset comprised 326 images with 3710 annotated objects, split at the sample level into training (70%), validation (15%), and testing (15%) sets. The YOLOv8n model achieved mean average precision (mAP@0.5) of 0.982 ± 0.015 across 5-fold cross-validation. Per-class AP exceeded 0.97 for five taxa, with *Trichomonas* achieving 0.952. Inference time averaged under 60 ms per image on a standard CPU. These results demonstrate that YOLOv8 provides accurate and efficient detection of diverse parasitic elements, supporting its potential as a clinical screening tool.

## Simple Summary

Microscopic examination of fecal samples is a fundamental method for diagnosing parasitic infections in dogs and cats, but traditional manual inspection is labor-intensive, time-consuming, and requires specialized expertise that may not always be available in busy veterinary practices. This study developed an automated detection system using a deep learning model called YOLOv8n to identify six types of parasites commonly encountered in veterinary medicine: egg-producing parasites (*Spirometra*, *Dipylidium*, hookworm, *Ascaris*) and single-celled parasites (*Giardia* cysts and *Trichomonas* trophozoites). Images were captured at three different magnifications (1000×, 2500×, and 10,000×) to evaluate detection performance across varying levels of morphological detail and field of view. The dataset comprised 326 microscopic images containing over 3700 individually annotated parasitic elements. The YOLOv8 model achieved high accuracy for five parasite types and demonstrated robust performance even for the smallest and most challenging targets, with detection speeds under 60 milliseconds per image on a standard computer. The 2500× magnification was identified as the most practical choice for routine screening, offering an optimal balance between fine detail and efficient sample coverage. The 10,000× magnification proved valuable for confirming ambiguous cases requiring detailed morphological examination. This automated tool has the potential to assist veterinarians by reducing manual screening time, improving diagnostic consistency, and ultimately supporting better healthcare outcomes for companion animals through faster and more reliable parasite detection.

## 1. Introduction

Pet health monitoring is an important part of modern veterinary practice, and early disease detection can substantially enhance therapeutic efficacy and reduce infection transmission risks [1,2]. Among the diagnostic methods, identifying parasitic infections is crucial for animal healthcare, protecting both pet health and preventing zoonotic transmission [3]. The detection of parasite ova in fecal samples serves as a primary diagnostic marker for such infections [4,5]. Due to their small size (generally 40–100 μm), these parasitic elements can only be reliably identified using high-powered microscopy and validated diagnostic methods [6]. Traditional diagnostic approaches have primarily depended on manual microscopy for parasite detection [7], an approach that is labor-intensive and time-consuming, with diagnostic performance highly dependent on operator expertise, potentially leading to variability and reduced sensitivity in low-intensity infections [8,9]. Consequently, there is a growing need for automated high-throughput detection systems that can reliably identify parasitic elements within minutes to advance veterinary diagnostics.

The rapid advancement of computer vision and deep learning has positioned machine learning-based approaches as transformative solutions for automating complex image analysis, particularly in detecting microscale biological structures such as parasite eggs, cysts, and trophozoites [10,11]. Among deep learning architectures, Convolutional Neural Networks (CNNs) have demonstrated superior performance in both fine-grained image classification [12,13] and small object detection [14,15], making them particularly suitable for identifying parasitic elements in microscopic fecal samples.

The YOLO (You Only Look Once) family of single-stage detectors has shown remarkable efficacy in this domain due to its real-time processing capability and robustness in detecting small, densely clustered objects under varying imaging conditions [16,17]. As the latest iteration, YOLOv8 introduces architectural refinements including an optimized backbone network and enhanced feature fusion mechanisms, which have been reported to improve inference speed and detection accuracy in various computer vision tasks [18,19,20]. However, the extent of these improvements—such as specific latency reductions or mAP gains—can vary considerably depending on the dataset, imaging conditions, and target object characteristics [21,22]. Recent studies have demonstrated the potential of YOLOv8 in livestock and field settings [23,24]. For example, applied YOLOv8-based detection for gastrointestinal parasites in sheep [25], highlighting both the promise and the practical challenges of deploying deep learning models in real-world veterinary contexts, including variable sample quality, limited computational resources, and the need for robust model generalization across diverse farm environments. However, the effective adoption of such tools also depends on veterinarians’ evidence-based skills, which a survey of US and Canadian veterinary colleges found to be inconsistently taught due to overcrowded curricula and underutilized librarian resources [26].

In clinical practice, the intended role of such automated tools requires careful definition. The system described in this study is designed as an assistive diagnostic tool for rapid primary screening of fecal samples, rather than a replacement for human expertise. By flagging suspected parasitic elements and providing confidence scores, it aims to reduce manual screening time and improve diagnostic consistency, while leaving final confirmation—particularly for ambiguous or low-confidence cases—to trained parasitologists. This human-in-the-loop approach balances automation efficiency with clinical oversight, addressing both the throughput demands of high-volume laboratories and the accuracy requirements of definitive diagnosis. Despite the rapid progress of deep learning in microscopic image analysis, automated detection of parasitic elements remains challenging due to three critical factors: (1) significant morphological similarity among different parasite species, (2) extreme scale variations introduced by different microscopic magnifications, and (3) complex and noisy fecal backgrounds that obscure egg boundaries. Existing studies often focus on single magnification settings or limited parasite categories, which restricts their applicability in real clinical scenarios.

In this study, we present a systematic evaluation of the YOLOv8 object detection framework for multi-species parasitic element identification across three microscopic magnifications (1000×, 2500×, and 10,000×). While lower magnifications offer broader contextual information and higher magnifications reveal finer morphological details, the optimal balance for automated detection remains unclear. By systematically comparing model performance across these scales, we aim to identify the most effective magnification for routine screening and to assess the feasibility of high-magnification imaging for confirmatory diagnosis. By analyzing model convergence behavior, magnification-dependent performance, and class-wise detection characteristics, we reveal how image scale and contextual information jointly affect deep learning-based parasite egg recognition. Our results demonstrate that an intermediate magnification (2500×) provides an optimal balance between morphological detail and contextual completeness, enabling robust detection with high accuracy and real-time performance. This work not only validates YOLOv8 as an effective tool for microscopic parasite egg detection but also provides practical insights for optimizing imaging conditions and deployment strategies in automated parasitological diagnosis.

## 2. Materials and Methods

### 2.1. Overview of the Proposed Diagnostic Framework

**Figure 1:**
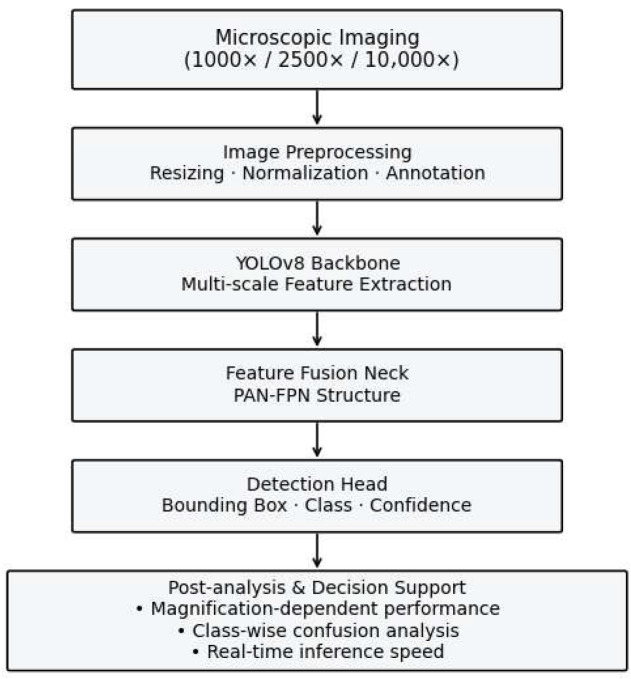
Overview of the YOLOv8-based multi-magnification parasitic element detection framework. Microscopic fecal images acquired at different magnifications are processed through a unified deep learning pipeline for automated egg detection, followed by performance analysis and confidence-guided diagnostic support.

To provide an overall understanding of the proposed diagnostic approach, Figure 1 illustrates the YOLOv8-based multi-magnification parasitic egg detection framework. The framework integrates microscopic image acquisition, deep learning-based object detection, and post-analysis to support automated veterinary parasitological diagnosis.

Microscopic images of fecal samples are acquired at three magnifications (1000×, 2500×, and 10,000×) to capture parasite elements with varying levels of morphological detail and contextual information. The images are first preprocessed through resizing and normalization to ensure compatibility with the YOLOv8 input requirements. Subsequently, multi-scale feature extraction is performed by the YOLOv8 backbone network, followed by feature fusion using a PAN-FPN neck to enhance the representation of small and densely distributed parasite elements.

The detection head outputs bounding boxes, class labels, and confidence scores for each detected egg. Finally, post-analysis is conducted to evaluate magnification-dependent performance, class-wise confusion characteristics, and inference efficiency. This design enables not only accurate automated egg detection but also confidence-guided diagnostic support, facilitating practical deployment in veterinary clinical settings.

### 2.2. Microscopic Image Acquisition and Dataset Construction

We assembled a microscopic image dataset featuring six clinically important parasitic taxa: *Spirometra* eggs, *Dipylidium* egg packets, hookworm eggs, *Ascaris* eggs, *Giardia* cysts, and *Trichomonas* trophozoites. Fecal samples were obtained from a total of 85 animals (approximately 50 dogs and 35 cats) and were provided by Henan Dake Teaching Instrument Co., Ltd. (Xinxiang, China). Samples from animals treated with anthelmintics within four weeks, or with insufficient volume or autolysis, were excluded.

Images were acquired using a microscope (DL-KY300, Delang Optical Instrument Co., Ltd., Shangrao, China) equipped with a 12-megapixel CMOS camera (SONY, 1/2.3″ sensor, 1.55 μm pixel size). The magnifications reported (1000×, 2500×, 10,000×) represent effective magnifications combining optical magnification (4×/0.1, 10×/0.25, and 40×/0.65 objectives; 10× eyepiece; 2.5× adapter) with 10× digital zoom applied during acquisition to maintain consistent 640 × 640 input resolution. Pixel-to-micron conversion factors, calibrated with a stage micrometer, are 0.155 μm/pixel, 0.062 μm/pixel, and 0.0155 μm/pixel, respectively.

All parasitic elements were annotated using the LabelImg tool (version 1.8.6) by two parasitologists following a standardized protocol: tight bounding boxes around egg shells/cyst walls/cell bodies (excluding flagella); partial objects (>50% visible) included overlapping objects annotated separately following COCO occlusion guidelines; debris explicitly excluded. Disagreements were resolved by a third senior expert to ensure quality, particularly for morphologically similar taxa (e.g., Hookworm vs. *Ascaris*).

The dataset comprises 326 images with 3710 annotated objects (Table S1). To compensate for lower object density at higher magnifications, image distribution favors higher magnifications: 65 images at 1000×, 98 at 2500×, and 163 at 10,000×. Each class contains approximately 610–660 objects (536 for *Trichomonas*), with validation and test sets each containing 30–40 objects per class—sufficient for reliable evaluation. To prevent data leakage, train/validation/test splitting (70%/15%/15%) was performed at the sample level (by animal) before any digital processing, ensuring all images from the same animal remained in a single subset.

### 2.3. YOLOv8-Based Object Detection Model

YOLOv8 represents an advanced deep learning architecture optimized for real-time object detection. Unlike conventional two-stage detectors that separately generate region proposals and perform classification, YOLOv8 unifies these processes into a single efficient framework, making it particularly suitable for time-sensitive applications such as microscopic parasite egg detection. As the latest iteration in the YOLO series, it demonstrates superior performance in both accuracy and speed, especially for small object recognition tasks. The model architecture comprises Backbone, Neck and Head, which are applied for feature extraction, multi-scale feature aggregation and bounding box regression and classification, respectively. Training optimization is achieved through a composite loss function. The YOLOv8 model was optimized using a composite loss function consisting of bounding box regression loss ($L_{box}$), classification loss ($L_{cls}$), and Distribution Focal Loss ($L_{dfl}$):

(1) $$L=L_{box}+L_{cls}+L_{dfl}$$

For localization, the Complete Intersection over Union (CIoU) loss was employed to improve bounding box regression. CIoU simultaneously considers the overlap area, center distance, and aspect ratio consistency between the predicted and ground truth boxes, thereby enhancing detection stability and accuracy. For classification, binary cross-entropy with logits was applied. Each class was treated independently using a sigmoid activation function, which is suitable for multi-category object detection tasks. Additionally, $L_{dfl}$ was introduced to refine bounding box coordinate prediction by modeling localization as a discrete probability distribution. This strategy improves boundary precision and contributes to more accurate parasite detection under microscopic imaging conditions.

### 2.4. Training Configuration

All experiments were conducted on a workstation equipped with an Intel Core i7-8700 CPU and 16 GB RAM, using PyTorch 2.0.1. We employed the YOLOv8n (nano) model from the Ultralytics implementation (version 8.0.0), initialized with weights pre-trained on the COCO dataset (transfer learning).

Image preprocessing: All original images were first converted to RGB format and normalized to the range [0, 1]. For 1000× and 2500× images, the entire field of view was directly resized to 640 × 640 pixels using bilinear interpolation. For 10,000× images, due to the reduced field of view, each original image was divided into overlapping tiles of 640 × 640 pixels with 20% overlap between adjacent tiles to ensure complete coverage of the sample area. Each tile was then independently processed by the model, and tiling was applied after sample-level splitting to prevent data leakage.

The model was trained using the following hyperparameters: SGD optimizer with momentum (β = 0.937), initial learning rate 0.01 with cosine annealing to 0.001, weight decay 0.0005, batch size 8, and 5 epochs with 3 warmup epochs (warmup initial momentum 0.8, warmup initial learning rate 0.001). Data augmentation included mosaic (probability 1.0), mixup (probability 0.5), HSV adjustments (hue ±0.015, saturation ±0.7, value ±0.4), random horizontal flips (probability 0.5), random scaling (±0.5), and random translation (±0.1). The composite loss function incorporated scale-specific weights [2.0, 1.5, 1.0] for small, medium, and large targets, combining CIoU for bounding box regression and binary cross-entropy for objectness and classification.

During inference, the following settings were applied: input image size 640 × 640 pixels, confidence threshold 0.25, IoU threshold (NMS) 0.45, and maximum detections per image 300. These thresholds were empirically determined based on validation set performance. Total training time for 5 epochs was approximately 2 h on the CPU-only workstation, with the best model selected based on lowest validation loss for subsequent test set evaluation.

### 2.5. Proposed Clinical Workflow

In routine veterinary practice, fecal samples processed by flotation or sedimentation are scanned under a microscope equipped with a digital camera. In the proposed workflow, the entire coverslip area is systematically scanned at 2500× magnification (approximately 100–120 fields per sample), with each field processed in real-time by the YOLOv8n model (<60 ms per field). Detected parasites are displayed as bounding boxes with confidence scores, flagging low-confidence detections (<0.6) for manual review. A summary report is generated with parasite types and estimated counts. For morphologically ambiguous targets, operators can acquire 10,000× images for confirmatory examination. This tiered approach balances screening efficiency with diagnostic accuracy while maintaining human oversight.

## 3. Results

### 3.1. Impact of Magnification on Detection Performance

**Figure 2:**
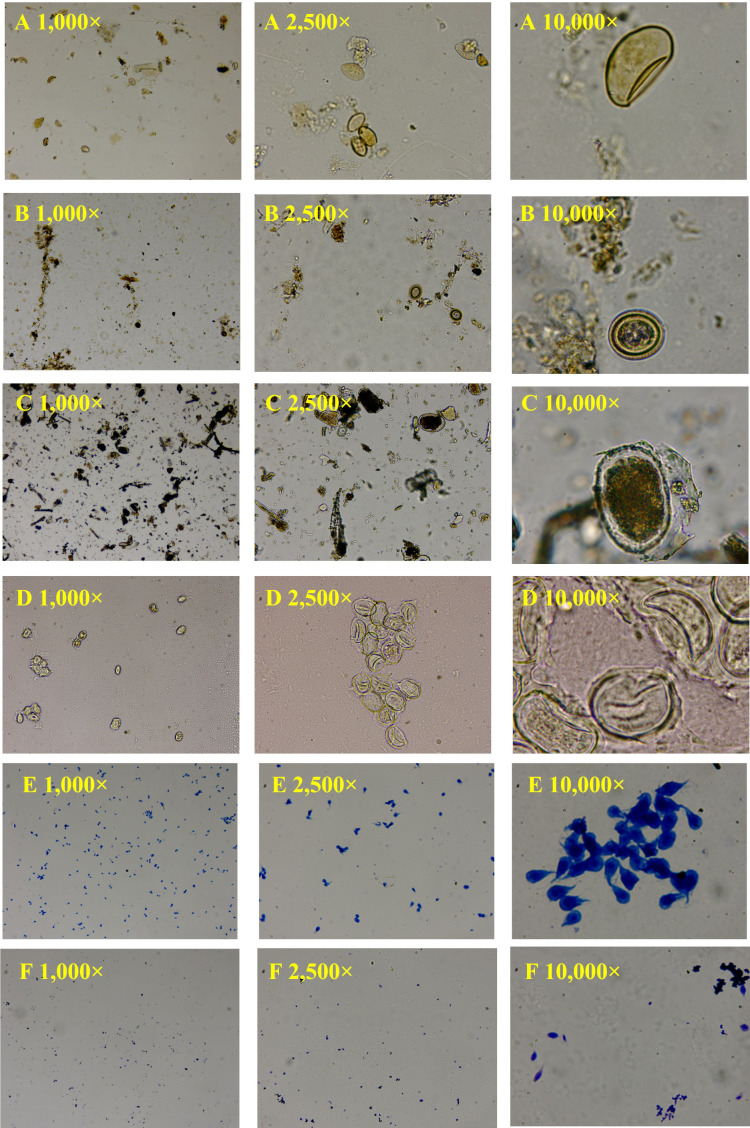
The parasite elements captured by microscopy with different magnifications: (**A**) *Spirometra* egg, (**B**) *Dipylidium* egg packet, (**C**) Hookworm egg, (**D**) *Ascaris* egg, (**E**) *Giardia* cyst, (**F**) *Trichomonas* trophozoite.

The magnification of microscopic images plays a pivotal role in the detection accuracy of small objects, such as parasite ova. In this study, parasite ova images were captured at three distinct magnifications: 1000×, 2500×, and 10,000× (Figure 2). These magnification levels were selected to evaluate how the level of image detail influences the performance of the YOLOv8 model in detecting and classifying parasite elements. At 1000×, images provide broad sample coverage but lower resolution, increasing risk of false positives/negatives for small or obscured targets. At 2500×, higher resolution maintains detailed views while preserving a reasonably broad field of view, striking an optimal balance for general identification. At 10,000×, fine morphological features are visible, but the field of view is substantially reduced—each 10,000× field covers only 1/16 of the area at 2500×.

To quantify practical trade-offs, we measured inference times on an Intel Core i7-8700 CPU (640 × 640 input). At 1000× and 2500×, where the entire field fits within a single tile, average inference times were 52 ± 4 ms and 58 ± 5 ms per image, respectively. At 10,000×, each raw image requires approximately 4 overlapping tiles to maintain coverage, resulting in 208 ± 16 ms per original field. At the sample level, assuming 100 fields at 2500× versus 1600 fields at 10,000× for equivalent coverage, total processing time increases from ~5.8 s to over 5 min—a 50-fold increase. These data confirm that while 10,000× preserves per-image discriminative power, its utility for primary screening is limited by the substantial increase in processing time. For high-throughput clinical applications, 2500× offers the optimal balance between diagnostic accuracy and operational efficiency.

### 3.2. Impact of Epochs on Model Performance

**Figure 3:**
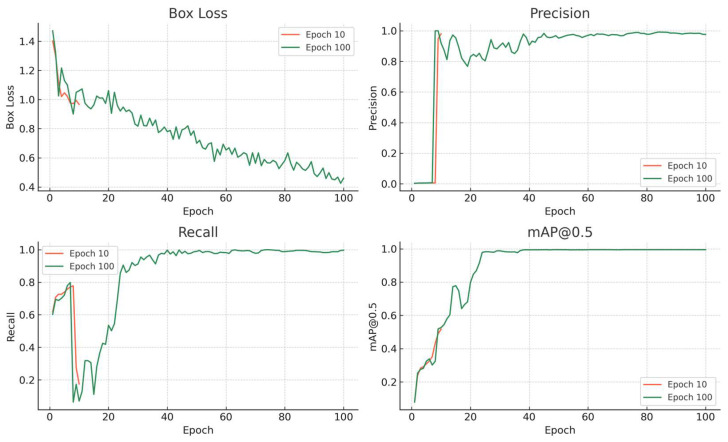
Training metrics (Box Loss, Precision, Recall, mAP@0.5) across 100 epochs, with markers at epochs 10 and 100 highlighting key progression points.

Data in Figure 3 presents a comparison of four critical training metrics—Box Loss, Precision, Recall, and mAP@0.5—for the YOLOv8 model trained up to 10 and 100 epochs (detailed epoch-wise metrics are provided in Table S2 in the Supporting Information). These metrics collectively provide a comprehensive view of the model’s learning progression and detection performance over time. The Box Loss curve shows a significant decline from the initial epochs to epoch 10, indicating rapid learning of object boundaries. Further reduction between epochs 10 and 100 suggests continued fine-tuning of bounding box predictions. Precision already exhibits reasonable performance by epoch 10, with further refinement by epoch 100, implying reduced false positives. Recall also improves notably from epoch 10 to epoch 100, as extended training enables better generalization and more comprehensive detection. The most impactful metric, mAP@0.5, rises steadily with training epochs, reaching near-saturation at 100 epochs—demonstrating the model’s convergence and readiness for deployment. Overall, these metrics demonstrate that while the model learns meaningful features early in training, extending to 100 epochs yields substantial gains in both localization and classification performance, justifying the computational cost for high-accuracy applications.

To further validate the robustness of the model trained for 100 epochs, we performed 5-fold cross-validation at the sample level (by animal). The dataset was randomly partitioned into five folds, with each fold containing approximately 20% of the samples. In each iteration, four folds were used for training and the remaining fold for validation, ensuring that images from the same animal never appeared in both training and validation sets. The model achieved consistently high performance across all folds, with mean mAP@0.5 of 0.982 ± 0.015, mean precision of 0.983 ± 0.012, and mean recall of 0.981 ± 0.014. The low standard deviations across folds confirm that our results are stable and not dependent on a particular train/validation split, mitigating concerns about potential data leakage or overfitting to a specific subset.

### 3.3. Effect of Microscopic Magnification on YOLOv8-Based Parasite Element Classification Performance

**Figure 4:**
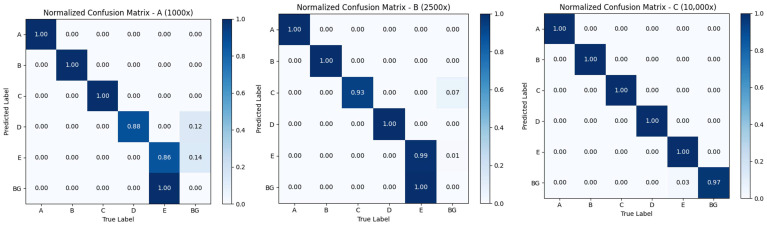
Normalized confusion matrices of YOLOv8-based parasitic element detection on the test set at three microscopic magnifications (1000×, 2500×, and 10,000×). Each row is normalized by the total number of ground truth instances for that class, with diagonal values representing per-class classification accuracy. Raw count confusion matrices are provided in Figure S1 in the Supporting Information. Classes A–E correspond to *Spirometra* eggs, *Dipylidium* egg packets, Hookworm eggs, *Ascaris* eggs, *Giardia* cysts, and *Trichomonas* trophozoites, respectively. BG denotes background.

Figure 4 presents the normalized confusion matrices of YOLOv8-based parasitic element detection on the held-out test set under three microscopic magnifications: 1000×, 2500×, and 10,000×. Each subfigure reflects the classification accuracy of different target types, encoded as A–F, corresponding to specific parasites (e.g., A: *Spirometra*, F: *Trichomonas*), with BG representing background. At 1000× magnification, the model achieves reasonable per-image classification performance across most categories, though noticeable misclassifications occur between certain targets and the background—particularly for *Giardia* (E) and *Ascaris* (D). When magnification increases to 2500×, the model yields the best overall results: per-image classification accuracy improves significantly, with minimal misclassification and balanced recognition across all classes. This suggests that 2500× provides an optimal balance between morphological detail and contextual clarity, enabling the model to distinguish subtle differences between species while maintaining sufficient field of view. At 10,000× magnification, a different trade-off emerges. While offering the highest feature resolution—which benefits recognition of fine details (e.g., *Trichomonas* (F) is well-recognized)—the substantially reduced field of view increases the imaging burden at the sample level, requiring more images to cover the same area. Notably, the model’s per-image discriminative power remains high, with background confusion minimized at this magnification, confirming that fine morphological details aid in distinguishing true targets from artifacts when fully visible.

These results demonstrate that for high-throughput automated screening, 2500× emerges as the most effective magnification, balancing detection accuracy with practical coverage efficiency. For confirmatory diagnosis of small or ambiguous targets (e.g., *Giardia* cysts, *Trichomonas* trophozoites), 10,000× remains valuable as a secondary, targeted imaging step.

### 3.4. Per-Class Performance and Error Analysis

We analyzed per-class precision, recall, and average precision (AP) at 2500× magnification (Table 1). The model achieved excellent results for most classes, with precision and recall above 0.97 and AP@0.5 exceeding 0.97 for classes A–E (*Spirometra*, *Dipylidium*, Hookworm, *Ascaris*, *Giardia*). Class F (*Trichomonas*) showed slightly lower but still robust performance (precision 0.96, recall 0.94, AP@0.5 0.952).

Systematic error analysis revealed three primary patterns. Target size accounted for 42% of errors, with small elements (*Giardia* cysts, *Trichomonas* trophozoites) frequently missed when adjacent to debris or partially obscured. Morphological similarity contributed to 28% of errors, primarily confusion between Hookworm and *Ascaris* eggs when diagnostic features were not clearly visible. Overlapping objects and background debris accounted for the remaining 30%, with clustered eggs merged into single detections and debris triggering false positives.

### 3.5. High-Magnification Parasitic Element Detection Using YOLOv8

**Figure 5:**
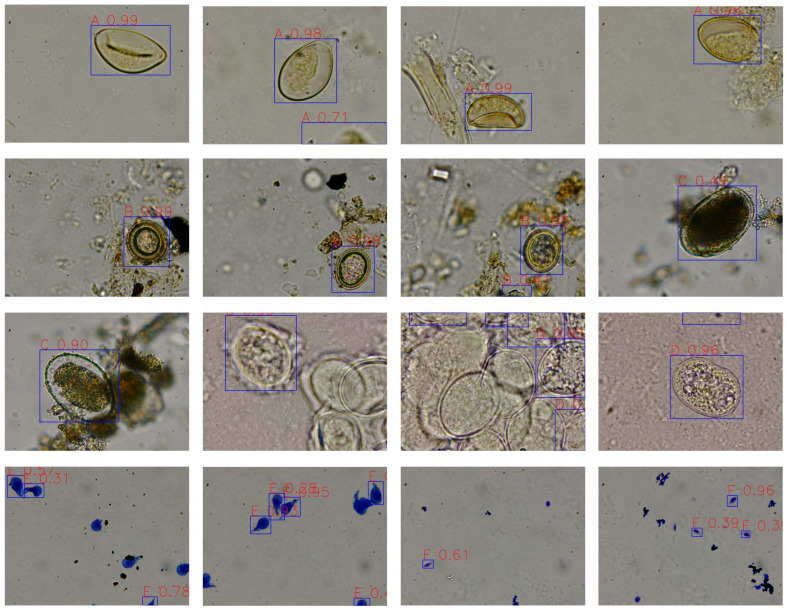
Microscopic images of parasitic elements detected by YOLOv8 at 10,000× magnification with confidence scores. (A) *Spirometra* eggs, (B) *Dipylidium* egg packets, (C) Hookworm eggs, (D) *Ascaris* eggs, (E) *Giardia* cysts, (F) *Trichomonas* trophozoites.

Although 2500× was identified as the optimal magnification for primary screening, high-magnification imaging at 10,000× remains clinically relevant for confirmatory diagnosis of small or morphologically ambiguous targets. Figure 5 presents representative detection results at 10,000× magnification on the test set.

The model successfully identified all six target taxa at 10,000× magnification. High confidence values (e.g., A 0.99, B 0.99, C 0.90, D 0.96) demonstrate the model’s ability to leverage fine morphological features for larger, well-defined targets. Lower confidence scores were observed for some instances of *Giardia* (E 0.28) and *Trichomonas* (F 0.39, 0.61), consistent with the error analysis: these targets are substantially smaller (10–20 μm vs. 40–100 μm for nematode eggs), making them more susceptible to partial occlusion and focal variations at extreme magnification. These results illustrate that while 10,000× enables detailed visualization of diagnostic features—beneficial for confirming species identity—it also demands optimal sample preparation and precise focusing. In clinical practice, a tiered approach is recommended: primary screening at 2500× followed by targeted 10,000× imaging for ambiguous cases.

## 4. Conclusions

This study systematically evaluated YOLOv8 for automated detection of parasitic elements in fecal samples across multiple magnifications, demonstrating high accuracy (Precision, Recall, and F1-score > 0.95 for most taxa) with real-time processing (<60 ms per image). A key finding is that 2500× magnification provides the optimal balance for primary screening, offering sufficient morphological detail while maintaining practical sample coverage. At this magnification, the model achieved near-perfect classification for five of six target taxa, with only Trichomonas showing slightly lower but still robust performance (AP@0.5 = 0.95). The lower confidence for smaller targets (*Giardia* cysts, *Trichomonas* trophozoites) reflects a multi-factorial trade-off rather than a simple resolution limitation, suggesting that future improvements could leverage multi-scale training or targeted data augmentation. While 10,000× magnification preserves per-image discriminative power for confirmatory diagnosis, its reduced field of view imposes practical trade-offs in coverage efficiency—leading us to propose a tiered workflow: primary screening at 2500×, followed by targeted 10,000× imaging for ambiguous cases. Study limitations include single-device testing, lack of external validation on independent datasets from different institutions or geographic regions, and unverified performance on edge devices or embedded systems (e.g., portable microscopes, mobile platforms) that may be used in field settings, as all experiments were conducted on a desktop PC. Future work should therefore focus on multi-institutional collaborations to collect diverse datasets, prospective clinical trials to validate real-world diagnostic accuracy, and deployment studies to assess model performance after optimization for resource-constrained environments.

## Figures and Tables

**Table 1:** Per-class performance metrics at 2500× magnification.

Class	Parasite	Precision	Recall	AP@0.5
A	*Spirometra* (eggs)	0.99	0.99	0.993
B	*Dipylidium* (egg packets)	0.99	0.98	0.987
C	Hookworm (eggs)	0.99	0.99	0.991
D	*Ascaris* (eggs)	0.99	0.99	0.992
E	*Giardia* (cysts)	0.98	0.97	0.976
F	*Trichomonas* (trophozoites)	0.96	0.94	0.952

## Data Availability

The original contributions presented in this study are included in the article/Supplementary Material. Further inquiries can be directed to the corresponding author.

## Supplementary Materials

The following supporting information can be downloaded at: https://www.mdpi.com/article/10.3390/vetsci13030257/s1, Figure S1: Confusion matrices of YOLOv8 models at different microscopic magnifications (1000×, 2500×, 10000×). A-F represents Egg of Spirometra, Dipylidium, Hookworm, Roundworm, Giardia and Trichomonas, respectively. BG means background, Table S1: Distribution of images and annotated objects across magnification levels and dataset splits.
