# Supplementary material for: Automated Detection of Parasitic Elements in Veterinary Fecal Samples Using a Deep Learning-Based Object Detection Framework
Source: Vet Sci. 2026 Mar 10;13(3):257. doi: 10.3390/vetsci13030257 (PMC13030632; doi:10.3390/vetsci13030257)
Supplement: Supplementary file 1 [file vetsci-13-00257-s001.zip › vetsci-4168441-supplementary.pdf]

Automated Detection of Parasitic Elements in Veterinary Fecal Samples using a Deep Learning–based Object Detection Framework

Jing Yang [1], Bo Yang [2], Qingxiang You [3], Zhenqing Li [3,*] and Yoshinori Yamaguchi[4]

1. Faculty of Engineering, Anhui Sanlian University, Hefei, 230000, China;
2. Engineering Research Center of Optical Instrument and System, Key Lab of Optical Instruments and Equipment for Medical Engineering, Ministry of Education, Shanghai Key Lab of Modern Optical System, University of Shanghai for Science and Technology, Shanghai 200093, China;
3. School of Computer Science and Information Engineering, Changzhou Institute of Technology, Changzhou, 213032, China
4. Department of Applied Physics, Graduate School of Engineering, Osaka University, Yamadaoka Suita-city, Osaka 565-0871, Japan

Correspondence: zhenqingli@163.com

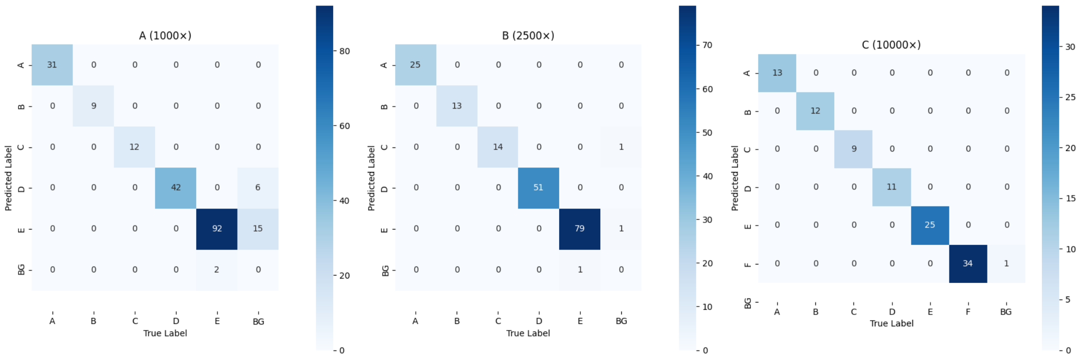

Fig.S1 Confusion matrices of YOLOv8 models at different microscopic magnifications (1000×, 2500×, 10000×). A-F represents Egg of *Spirometra*, *Dipylidium*, *Hookworm*, *Roundworm*, *Giardia* and *Trichomonas*, respectively. BG means background.

Table S1. Distribution of images and annotated objects across magnification levels and dataset splits.

Magnification	Split	Images	A	B	C	D	E	F	Total Objects
1000×	Training	46	108	104	112	106	102	90	622
	Validation	10	23	22	24	22	21	19	131
	Testing	9	24	23	25	23	22	19	136
2500×	Training	69	162	158	166	160	154	135	935
	Validation	14	34	33	35	33	32	28	195
	Testing	15	36	35	37	35	34	30	207
10000×	Training	114	180	176	184	178	172	150	1,040
	Validation	24	38	37	39	37	36	32	219
	Testing	25	39	38	40	38	37	33	225
Total		326	644	626	662	632	610	536	3,710

Table S2. Key training metrics from Epoch 10 to Epoch 100.

Epoch	Precision	Recall	mAP@0.5
10	0.9153	0.1521	0.4374
20	0.8472	0.5227	0.7957
30	0.9218	0.9159	0.9821
40	0.9147	0.9862	0.9918
50	0.9359	0.9951	0.9941
60	0.9461	0.9893	0.9946
70	0.9258	0.9872	0.9948
80	0.9875	0.9813	0.9949
90	0.9937	0.9935	0.9935
100	0.9871	0.9991	0.9949
